# Rethinking scientific diaspora engagement models in fragile contexts: the Venezuelan case

**DOI:** 10.3389/frma.2026.1864434

**Published:** 2026-07-29

**Authors:** Branislav Pantović, Luisa F. Echeverría-King, Kleinsy Bonilla, Daniela Moreno Garcia

**Affiliations:** 1 Universidad Simón Bolívar, Barranquilla, Colombia; 2 DiploCientifica, Viedma, Argentina; 3 Observatorio Económico Sostenible, Universidad del Valle de Guatemala, Guatemala City, Guatemala

**Keywords:** diaspora engagement, international cooperation, knowledge circulation, migration, science diplomacy, scientific diasporas, STI, Venezuela

## Abstract

The Venezuelan case demonstrates the limitations of traditional diaspora engagement models, which typically assume stable national science, technology, and innovation systems as well as functional state–diaspora relations. Since these models do not adequately address diaspora engagement in fragile contexts, this article argues that different approaches are necessary. The analysis indicates that the existing literature lacks both an awareness of fragility and a model of diaspora engagement tailored to cases such as Venezuela. Drawing on research on scientific diasporas, science diplomacy, knowledge circulation, and science policy, this study analyzes how engagement is conceptualized, enacted, and constrained. The argument is substantiated by qualitative evidence from focus groups with members of the Venezuelan scientific diaspora, who described the extended institutional crises and periods of dispersion that Venezuela faces. The study identifies key challenges, experiences, and expectations, emphasizing the role of diasporas as non-state actors who could connect national and international scientific agendas and influence development. The article concludes by recommending a flexible, context-sensitive approach to scientific diaspora engagement and provides policy recommendations.

## 1 Introduction

Scientific knowledge is increasingly influenced by geopolitical competition, conflict, and strategic positioning (Echeverría King et al., 2024). In this context, international cooperation remains essential and drives model innovations that address the complexities of a rapidly changing world. Moreover, scientific diasporas have emerged as a particularly relevant form of transnational resource capacity, with the potential to connect institutions, circulate knowledge, and support development. The policy document *Bridging Knowledge Frontiers: A Comparative Analysis of International Strategies and Methodologies to Engage Scientific Diasporas and Foster Transnational Collaboration* (Echeverría-King et al., 2025) mapped international experiences, identifying recurring patterns, lessons, and enabling factors across cases. This analysis reveals an expanding range of engagement models, including scientific networks, digital platforms, short-term mobility schemes, and inter-institutional collaboration. Notably, the most effective and sustainable experiences tend to rely on predictable governance frameworks, stable funding, and a high degree of institutional continuity. These models are typically established in environments characterized by strong institutional capacity, clear state coordination, and consistent policy implementation. However, such conditions are often absent in contexts where diaspora engagement is most urgently required.

Although there is growing consensus on the strategic value of scientific diasporas and an expanding repertoire of engagement activities, the field still lacks consolidated knowledge about what works, under which institutional conditions, and how impact should be assessed. The literature reviewed by Bonilla et al. (2023) and also by Jarquín-Solís (2022) suggest that diaspora engagement serves as a science diplomacy mechanism for bridge-building, through which countries connect with the expertise, networks, and collaborative potential of their scientific communities abroad. The literature also shows that the field has produced some normative claims and practical experiments, yet much less robust comparative knowledge of the institutional conditions, governance arrangements, and evaluative tools that make diaspora-engagement models sustainable and appropriate. Many models might fail in the case of institutional fragility because they assume institutional continuity that does not exist. In contexts marked by crisis, political volatility, or contested governance, the institutional assumptions underlying many successful international models may prove ineffective. Consequently, copying those engagement models often brings partial and discontinuous results, overly reliant on individual diaspora initiatives, voluntary cooperation, and/or short-term projects.

The Brazilian case study notes that national literature converges on the diaspora's potential, the “*lack of consistent public policies*,” and the need to “*institutionalize engagement mechanisms*” that are inclusive and sustainable, while the regional literature identifies a “*lack of systematic data on qualified diaspora populations*,” together with “*fragile institutional frameworks and unclear regulatory environments*,” as recurring obstacles to durable linkage mechanisms (Ferreira and Gimenez, 2025, pp. 6, 8). The Colombian case study sharpens this disparity by stating that “*there is no institutional or political instrument that evaluates the effectiveness of both science diplomacy and the scientific diaspora*” in relation to public policies objectives (Piñeros Ayala, 2025, p. 18). Accordingly, diaspora engagement might risk “*fragmentation and underperformance*” without a strong policy mechanism, and explicitly call for “*rigorous monitoring and evaluation systems with clear indicators and continuous feedback mechanisms*.” (Echeverría-King et al., 2025, p. 7). This concern is especially salient in fragile institutional settings.

Therefore, the question guiding this article is: What conceptual and policy modifications are applicable to make scientific diaspora engagement models viable in the Venezuelan context, which is defined by institutional precarity and a prolonged crisis? The Venezuelan case should be understood in this article as unique because its specificity lies in the scale, duration, and combination of mass scientific emigration, weakened STI institutions, damaged trust, limited absorptive capacity, and the absence of stable mechanisms for diaspora engagement. The specificity of the Venezuelan case should first be situated within the broader scale of national displacement: according to UNHCR, “Nearly 7.9 million people have left Venezuela in search of protection and a better life,” and “the majority—more than 6.9 million people—are hosted in Latin American and Caribbean countries” (UNHCR, 2025). In 2020, at the scientific level, Diez et al. (2020) stated that “Venezuela has lost through migration 16% of its research workforce” while also finding that even among researchers not planning to return, “there was evidence of their desire to collaborate with local partners in academic, professional, or business organizations and to engage in community work.” These conditions call for conceptual and policy adaptations that strengthen institutional capacity, enable hybrid forms of STI participation, improve coordination across actors, and make engagement mechanisms more credible and sustainable. The answer presents viewpoints and interpretations that draw on advances in the specialized body of literature, as well as on arguments grounded in primary data collected directly from members of the Venezuelan scientific diaspora.

The argument is informed by three 90-min focus groups with representatives of Venezuelan scientific diaspora networks selected to reflect disciplinary, geographic, and gender diversity (*n* = 11). Table 1 presents an overview of the background of the participants, showing diversity in their regions of residence (territory), gender inclusive, fields of knowledge and professional/career stage.

**Table 1:** Focus groups participants—a brief overview.

Focus group	Participant	Gender	Diasporic destination	Profile overview—field of research—stage in career development
1	Participant 1	M	USA/North America	Computational biology, bio-stadistics, health sciences—Early career
	Participant 2	M	USA/North America	Computer engineering and political science—Data science—Early career
	Participant 3	M	Belgium/Europe	Chemical engineering, plant design, simulations early career
	Participant 4	F	USA/North America	Educational biology, genetics education—Senior career
2	Participant 5	F	Brazil/South America	Sociology, science and technology policy—Mid-career
	Participant 6	F	Brazil/South America	Health science, neurosciences—Mid-career
	Participant 7	F	Italia/Europe	Philosophy and applied physics—Senior career
3	Participante 8	M	Spain/Europe	Biology and health sciences, biosafety—Senior career
	Participante 9	M	Colombia/South America	Physics, advanced computing—Senior career
	Participante 10	F	Colombia/South America	Ethnobiology, molecular plan biology—Senior career
	Participante 11	M	Spain/Europe	Sociology, international mobility and diaspora studies—Senior career

### 1.1 Methods and empirical material

This perspective article draws on qualitative empirical material collected through three focus groups with members of the Venezuelan scientific diaspora. The focus groups were designed to explore how diaspora researchers and professionals understand their current forms of engagement, the barriers they face, and the types of instruments that could enable more effective and sustainable collaboration with scientific ecosystems in Venezuela and Latin America. Participants were purposefully selected to reflect diversity in disciplinary backgrounds, geographic locations, professional trajectories, and experience in scientific, academic, technological, or organizational activities linked to the Venezuelan diaspora.

The focus groups brought together 11 participants currently based in different regions, including North America, Europe, and Latin America. They included researchers, university professors, STEM professionals, members of diaspora organizations, and individuals with experience in scientific networks, capacity-building initiatives, and knowledge-transfer activities. The conversations followed a semi-structured guide organized around five main topics: existing diaspora engagement activities, the needs and interests of the organized scientific diaspora, possible mechanisms for international funding agencies, institutional and operational barriers, and recommendations for future instruments or programs. All discussions were conducted under conditions of confidentiality, and the information is presented anonymously.

The empirical material was analyzed through thematic coding, combining categories derived from the literature on scientific diasporas with themes that emerged inductively from participants' narratives. The analysis focused on recurrent patterns across the three focus groups, including willingness to contribute, weak institutional coordination, limited sustainable financing, reliance on informal networks, barriers to recognition and mobility, the limited viability of permanent return, and the need for hybrid mechanisms of knowledge circulation. Given the small, purposive nature of the sample, the findings are not presented as statistically representative of the Venezuelan scientific diaspora as a whole. Rather, they provide in-depth qualitative insights into trajectories, lived experiences, and policy expectations that help ground the article's argument about the need for context-sensitive models of diaspora engagement in fragile institutional settings.

## 2 Rethinking diaspora engagement

The literature on scientific diasporas has progressively shifted from interpreting highly skilled emigration primarily as brain drain to understanding diaspora communities as transnational actors involved in knowledge circulation, scientific cooperation, and development. Meyer and Brown-Luthango reframed scientific diasporas as an alternative way of thinking about the brain drain problem, emphasizing networks and transnational linkages rather than only return (Meyer and Brown-Luthango, 1999). This perspective was further developed through the literature on diaspora knowledge networks, which highlighted how expatriate professionals can contribute to their countries of origin through sustained intellectual, institutional, and technological connections (Meyer and Wattiaux, 2006). More recent contributions continue in this direction. Bonilla et al. argue that scientific diasporas can function as bridges between scientifically lagging and scientifically advanced nations, while Jarquin-Solis et al. show how diaspora experiences can generate policy lessons for research and development strategies in countries of origin (Bonilla et al., 2023; Jarquin-Solis et al., 2022). These perspectives show a broad conceptual consensus: scientific diasporas should not be treated only as populations to be repatriated, but as transnational communities capable of generating value through collaboration, mediation, and knowledge exchange.

This reconceptualization is especially important in Latin America and the Caribbean, where a growing body of literature has documented diverse forms of diaspora engagement beyond permanent return. The Argentine case study reinforces this argument and demonstrates that Law 26.421 established RAICES as a state policy, thereby ensuring its continuity beyond a single administration. Moreover, RAICES professionalized and formalized diaspora networks abroad, creating channels through which scientists could “foster connections between the diaspora and the scientific community in Argentina” and act as advisors and promoters of cooperation with host countries (Pombo, 2025).

Studies from Mexico, Guatemala, Costa Rica, and Colombia emphasize the role and challenges of scientific diasporas in building academic networks, facilitating international collaboration, supporting higher education, and contributing to science diplomacy and STEAM capacity-building (Gómez-Flores et al., 2022; Bonilla et al., 2022; Jarquin-Solis et al., 2022; Avendano-Uribe et al., 2022). The policy document by Echeverría-King et al. (2025) especially systematizes these developments through a comparative matrix of international experiences, showing that current engagement models increasingly rely on hybrid mechanisms such as digital platforms, short-term mobility, scientific networks, and inter-institutional coordination rather than on physical return alone (Echeverría-King et al., 2025). National policy studies produced within that same broader project confirm this trend: The Argentine case underlines the value of institutionalized programs, while the Mexican, Brazilian, and Colombian studies show that states are increasingly exploring formal and informal ways to connect with highly skilled communities abroad (Pombo, 2025; Hernández Mondragón and Cuapio, 2025; Ferreira and Gimenez, 2025; Piñeros Ayala, 2025). As a whole, the literature demonstrates that the field has become richer both conceptually and instrumentally, with a growing repertoire of policy tools and organizational arrangements.

Scientific diasporas are understood as actors of science diplomacy insofar as they connect national scientific priorities with international networks, facilitate cross-border cooperation, and create channels for technical and institutional exchange (Prieto and Scott, 2022). This is especially evident in emerging economies, where science diplomacy aims to address development objectives and bridge gaps in STI (Echeverría King et al., 2024). In the Colombian case study, Piñeros Ayala argues that a robust relationship between the state and its scientific diaspora requires a “*clear, continuous, and integrated scientific diplomacy strategy*”, while stressing that current efforts remain too intermittent and shallow to build durable links between public institutions and scientific communities abroad (Piñeros Ayala, 2025, pp. 2, 17). Pantović and Michelini (2018) examine the Serbian context, illustrating how the scientific diaspora functions as an actor in science diplomacy by mobilizing expertise and transnational networks to increase international visibility and support diplomatic engagement. In this context, the Ministry of Foreign Affairs identified the diaspora as a strategic partner in advancing its initiatives. More broadly, the literature on scientific diasporas shows that organized diaspora communities can strengthen science diplomacy by serving as bridges for cooperation projects, knowledge exchange, and technology transfer between countries of origin and their communities abroad (Bonilla et al., 2023, p. 3).

The literature is far less conclusive about the institutional and governance conditions that sustain models, especially in fragile settings. Here, policy studies point repeatedly to discontinuity, fragmentation, and weak evaluation. “Formal frameworks are necessary,” but they must be matched by “mechanisms for institutional integration,” because otherwise “symbolic recognition risks remaining superficial” (Hernández Mondragón and Cuapio, 2025). Ferreira and Gimenez note the scarcity of consistent public policies and the need to institutionalize engagement mechanisms, while also identifying the absence of systematic mapping, fragile institutional frameworks, and unclear regulatory environments as recurring barriers in the region (Ferreira and Gimenez, 2025). The Mexican case study similarly shows that diaspora governance often operates as a fragmented mosaic rather than as a coherent system, depending heavily on individual initiative and sporadic support (Hernández Mondragón and Cuapio, 2025). Likewise, Piñeros Ayala explicitly notes the absence of policy instruments capable of evaluating the effectiveness of scientific diplomacy and diaspora engagement in relation to public objectives in Colombia (Piñeros Ayala, 2025). Namely, diaspora engagement efforts risk fragmentation and underperformance without strong policy frameworks, sustained funding, and rigorous monitoring and evaluation systems (Echeverría-King et al., 2025).

There is the gap in literature: while the field has generated important normative arguments, most models explain how to engage diasporas under relatively stable conditions, but not how to do so when institutions are weak and crisis-affected, trust is damaged, and collaboration depends on informal networks. Comparative evidence from Latin America and beyond suggests that no single “best model” of diaspora engagement exists; rather, what travels across cases is a set of recurring adaptations that make collaboration viable under different institutional conditions.

## 3 Insights from the Venezuelan case

The Venezuelan case provides empirical grounding for the article's central argument. The specificity of the Venezuelan case lies in the particular combination, intensity, and duration of several overlapping dynamics: large-scale emigration, prolonged weakening of the national STI system, institutional fragmentation, erosion of trust between diaspora actors and state-level institutions, limited absorptive capacity in universities and research centers, and the strong reliance on informal or self-organized diaspora initiatives.

The focus-group material suggests that the Venezuelan scientific diaspora is not only a dispersed population of highly skilled professionals, but also a transnational knowledge ecosystem whose potential depends on the conditions under which collaboration can be organized, financed, and sustained. The thematic coding of the focus-group material identified four recurrent themes: willingness to contribute despite weak institutional channels; reliance on informal networks and voluntarism; the limited viability of permanent return and the need for hybrid knowledge circulation; and unequal trajectories among diaspora researchers depending on seniority, career stage, and host-country conditions. This helps show how the Venezuelan case differs not because scientific emigration itself is unprecedented, but because engagement with the diaspora occurs under particularly fragile and politically complex conditions.

### 3.1 Willingness to contribute with weak institutional articulation and lack of sustainable funding

The Venezuelan scientific diaspora was consistently described by participants as a highly skilled, widely distributed community that is still eager to work with organizations and actors associated with Venezuela. The diaspora was referred to as one of the nation's most important strategic assets throughout the talks, mainly due to its global networks, accumulated experience, and ability to support scientific advancement from overseas. As one participant stated, “*we see the diaspora as an enormous asset, as Venezuela's best international reserve*.” However, focus-group participants identified the main bottlenecks to engagement as low institutional coordination, weak sustainable financing, limited infrastructure, reliance on informal networks, and insufficient public and regional architecture for articulation. In this sense, the central problem is sustained mechanisms to link dispersed expertise to scientific, institutional, political and regional agendas.

### 3.2 Informal networks and voluntarism

Collaboration was often described as depending more on individual initiative, trust-based relationships, and personal contacts than on stable public mechanisms or coordinated national strategies. In this context, the Venezuelan scientific diaspora functions as a self-organized network of cooperation and a transnational knowledge ecosystem with notable social resilience. However, its capacity for long-term scaling is constrained by the absence of strategic integration, coordination tools, and recognition. One participant summarized this situation by stating that “*there are no institutions to do this*,” while another explained that much of the existing collaboration depends on “*former colleagues or friends*.” Overall, findings suggest that *ad hoc* efforts and informal relationships currently matter more for engagement than robust infrastructures of connection. While this supports globally distributed Venezuelan knowledge, this model is also characterized by overload, informality, and discontinuity. Funding appeared as a particularly recurrent concern, expressed very directly by one participant: “*if you ask me what the diaspora needs, it is money*.”

### 3.3 Return is limited; hybrid knowledge circulation is more realistic

Participants also emphasized that many members of the diaspora remain interested in contributing to Venezuela, although permanent return is often viewed as unrealistic due to professional consolidation abroad and uncertainty in the domestic context. One participant captured this clearly: “*many scientists are already well established in other countries and will not return to Venezuela, but they are still Venezuelan*.” Therefore, structural obstacles like inadequate funding, disjointed coordination, weakened scientific institutions, and the lack of updated databases, customized calls for collaboration, and systematic university linkage programs were the most common themes rather than symbolic belonging or expectations of repatriation. This finding suggests that diaspora engagement should not be designed exclusively around physical return, but around flexible mechanisms of knowledge circulation. For instance, remote collaboration, short-term visits, co-supervision, virtual teaching, mentoring, project-based engagement, and participation in international networks might be more appropriate forms of contribution under current conditions.

### 3.4 Unequal trajectories

The focus groups also showed that the Venezuelan scientific diaspora is not homogeneous. Senior researchers often referred to consolidated networks, previous institutional experience, and ongoing international collaborations. Early-career researchers, by contrast, face greater difficulties accessing funding, recognition, and stable opportunities for scientific insertion. As one participant noted, “*support is needed for those who are just starting*.” This suggests that policies toward the Venezuelan scientific diaspora should not assume a single profile or pathway, but should distinguish between senior and early-career researchers, between different host-country conditions, and between forms of engagement that require different levels of institutional support.

### 3.5 Diaspora-oriented science diplomacy

It was also clear that science diplomacy is essential for connecting the Venezuelan scientific diaspora with national, regional, and global scientific ecosystems. Participants mentioned the need for bridge-building mechanisms to connect academic institutions, governments, civil society, and diaspora organizations. From an analytical perspective, these results divert attention from the shortage of talent overseas to shortcomings in governance and coordination. They recommend rethinking Venezuelan diaspora engagement through more adaptable models of information sharing, hybrid forms of involvement, trust-based cooperation, and distinct mechanisms sensitive to both national development requirements and crises.

## 4 Implications for fragile institutional contexts

The Venezuelan case suggests that diaspora engagement in fragile institutional contexts should be judged not by the existence of “good practices” in the abstract, but by whether states and institutions can build minimum conditions for credible articulation. Across the three focus groups, senior researchers tended to describe diaspora engagement through consolidated professional networks, previous institutional roles, and existing international collaborations. By contrast, less institutionally established researchers emphasized instability, lack of recognition, limited access to funding, and difficulties entering formal scientific circuits after migration. Differences also appeared by host context: participants based in North America and Europe often emphasized regulatory, financial, and grant-management barriers linked to working with Venezuela, while those based in Latin America stressed institutional fragility, limited funding, and the difficulty of building regional scientific capacity under precarious conditions.

Focus-group participants did not include in the main problems the lack of willingness, but all of them rather described the absence of credible and sustained mechanisms through which dispersed expertise can be linked to scientific, institutional, and regional agendas. Moreover, focus-group results suggest that there is the need to adapt engagement instruments to context—especially where institutions are fragile, continuity is uncertain, and diaspora collaboration depends as much on trust, visibility, and adequate infrastructures as on formal policy design.

These findings speak directly to the questions raised in the literature review. As the literature on scientific diasporas and diaspora knowledge networks has argued, highly skilled migration can be reframed from brain drain to brain circulation when expatriate scientists remain connected through networks, collaboration, and knowledge exchange (Meyer and Brown-Luthango, 1999; Meyer and Wattiaux, 2006). More recent work has further shown that scientific diasporas can operate as bridges for development and science diplomacy, contributing to research capacity, transnational collaboration, and international articulation without necessarily returning permanently (Bonilla et al., 2023; Jarquin-Solis et al., 2022; Prieto and Scott, 2022; Pantović and Michelini, 2018). However, the Venezuelan case shows that this potential depends on more than the existence of highly qualified talent abroad or willingness to collaborate. Engagement models also require minimum conditions of trust, institutional coordination, absorptive capacity, stable funding, and credible channels of articulation. Taken together, the most effective engagement strategies with the scientific diaspora combine “hybrid, flexible, inter-institutional, and digital participation methods” (Echeverría-King et al., 2025, p. 6).

In this sense, the Venezuelan case is presented as a context that exposes the limits of models that assume relatively stable institutions, predictable funding, and functional state–diaspora relations. There must be highlighted that the Venezuelan case combines several overlapping dynamics: prolonged economic and institutional deterioration, severe underfunding of the scientific and university system, weakened research infrastructure, erosion of academic autonomy, politicization of institutional spaces, and constraints that have affected trust between researchers, universities, and state actors.

Thus, in the Venezuelan context, scientific diaspora engagement models need to move toward context-sensitive arrangements that treat collaboration as a strategy. We highlight that, in fragile settings, one of the first major steps is to recognize the potential of the diaspora through official decisions that create institutional conditions to empower, connect and sustain flexible forms of collaboration. At the same time, these efforts could be aligned with specific challenges and with Venezuela's needs and priorities. The effectiveness of diaspora engagement, therefore, depends on coordinated action across formal institutions and diaspora-led initiatives, rather than on isolated initiatives or symbolic appeals.

We recommend several key policy adaptations, outlined below, to improve diaspora engagement while taking Venezuelan institutional contexts into account. Following adaptations have three priorities: building minimum institutional credibility, creating infrastructures of articulation, and recognizing the diaspora as a strategic transnational actor.

- **Build and prioritize trust and continuity:** pursue the highest feasible level of institutionalization—legal where possible, programmatic and inter-institutional where necessary—with clear rules, stable programs, and predictable support so that engagement is seen as credible and worth sustaining over time. The focus groups imply that policies must invest in domestic absorptive capacity. Likewise, improve coordination among ministries, STI agencies, universities, embassies, and diaspora organizations to give engagement greater coherence plus continuity.
- **Build an infrastructure of connections:** create visible and durable mechanisms—such as directories, web platforms, and matchmaking tools—that make collaboration easier and support cross-border scientific linkages. The focus groups stressed that Venezuelan institutions commonly lack the capacity to receive and sustain international collaboration. In other words, diaspora policy without institutional strengthening at home would have a limited effect. Additionally, enable short-term, remote, and flexible modes of engagement so diaspora scientists can contribute without returning permanently. That means creating registries, platforms, calls, and formal channels instead of leaving engagement to friendship and improvisation. Focus groups especially mentioned that policy should incorporate hybrid and digital models more seriously.
- **Combine financial and non-financial incentives:** pair funding with recognition, visibility, access to networks, and institutional legitimacy to make engagement more attractive and sustainable. The focus groups mentioned different options and tailored instruments such as binational or multilateral grants, seed funds for joint research, micro-grants for mentorings and virtual training, support for short mobility, calls for remote collaborative laboratories, and funding for thematic networks.
- **Support networks without co-opting them:** recognize diaspora networks as legitimate partners while protecting their autonomy and their ability to sustain collaboration from the bottom up.
- **Reframe the diaspora as an actor, not just a resource:** treat diaspora scientists as transnational actors who can connect institutions, circulate knowledge, and shape collaborative agendas through co-design, representation in advisory spaces, and recognition as agenda-setting partners. Namely, the focus groups imply a need for diaspora-oriented science diplomacy as scientific diasporas should be understood not simply as pools of talent abroad, but as strategic transnational actors. This idea emerges from focus-group observations suggesting that diaspora networks can make science diplomacy and international STI cooperation more viable by building bridges across institutions and linking national scientific agendas with international ones.

These recommendations are intended for a diverse set of stakeholders rather than a single entity or solely state institutions. They target a multi-actor ecosystem that includes self-organized diaspora networks, Venezuelan universities and research centers, state-level STI and foreign policy actors where applicable, international cooperation agencies and funders, regional scientific organizations, and civil society or private-sector participants engaged in knowledge circulation. Specifically, diaspora networks may function as autonomous transnational knowledge brokers with distinct capacities and expectations. Universities and research centers can serve as nodes of articulation and interlocutors, but they require support to enhance their capacity to absorb and sustain collaboration, given diminished absorptive capacity. State-level actors are positioned to provide legitimacy, long-term continuity, and coordination, but they operate within a context of institutional fragility and limited trust, which constrains continuity. International funders and regional organizations can act as enabling intermediaries by supporting flexible, low-risk, and hybrid forms of collaboration, and are particularly important for establishing credible mechanisms for articulation and financing.

Approaching diasporas through the proposed policy adaptations redefines the power and viability of diaspora engagement under conditions of systemic fragility. The objective is not only to generate benefits for local scientific systems, but also to widen the scope for action available to countries facing asymmetries in their STI positioning, and integration into global knowledge systems. Ultimately, the Venezuelan case suggests that effectively engaging the scientific diaspora depends on how countries confront crises, protect scientific sovereignty, use science diplomacy to broaden channels of international articulation, and enhance their room for maneuver within an unequal global knowledge order.

## 5 Conclusions

Findings address the questions raised in the literature review about the institutional conditions under which scientific diaspora engagement models can become viable and sustainable. The literature on brain circulation, diaspora knowledge networks, science diplomacy, and transnational collaboration has shown that scientific diasporas can contribute to development without necessarily returning permanently to their countries of origin. However, the Venezuelan case shows that this potential depends on more than the existence of highly qualified talent abroad or willingness to collaborate. Engagement models also require minimum conditions of trust, institutional coordination, absorptive capacity, stable funding, and credible channels of articulation. In this sense, the Venezuelan case is not presented as exceptional in absolute terms, but as a context that exposes the limits of models that assume relatively stable institutions, predictable funding, and functional state–diaspora relations.

This article argues that engaging the scientific diaspora should be understood not as a secondary or symbolic policy option, but as a strategic question of science diplomacy, scientific sovereignty, and institutional STI capacity under conditions of precarity and crisis. In contexts such as Venezuela, where prolonged instability, fragmented governance, and interrupted scientific trajectories limit forms of international STI articulation, the diaspora can serve as a vector for sustaining cross-border engagements, linking national priorities to international networks, and converting externally located expertise into a durable resource for collective action and resilience. At the same time, this article's findings make clear that such actions cannot rest on voluntarism alone. It depends on the construction of credible and sustained mechanisms through which knowledge, trust, and institutional coordination can circulate across borders.

The focus-group findings consistently point to the importance of policy and institutional design, and the message is fairly consistent: the problem is not a lack of talent or willingness, but the absence of a public and institutional architecture capable of identifying, connecting, financing, and sustaining collaboration with the diaspora.

For this reason, the viability of diaspora engagement in the Venezuelan case requires a shift from standardized models based on transferable good practices toward more context-sensitive arrangements. Nevertheless, no single institution/actor can address this challenge in isolation. The government can provide direction, legitimacy, and continuity; international funding agencies and cooperation bodies can create adaptive instruments and sustained support; universities and research centers can function as nodes of articulation and co-production; and diaspora networks can mobilize knowledge, trust, and transnational reach. What is required, therefore, is a shared commitment grounded not in symbolism, but in institutional and political will. In other words, the real question is not whether highly qualified expertise abroad has potential, but whether states and institutions are willing and able to make cooperation viable under conditions of fragility and to treat the diaspora as a sustained, legitimate, and strategic resource for development, international agenda-setting, and greater margins of maneuver within unequal global knowledge systems.

Beyond its immediate relevance to Venezuela, this perspective underscores that fragile institutional contexts demand a different approach to engaging the scientific diaspora. The article argues that such engagement should be evaluated according to the presence of minimum conditions for articulation: trust and continuity, visible infrastructures of connection, institutional anchoring of participation, and flexible mechanisms that can sustain transnational collaboration over time, while recognizing the diaspora as a strategic actor rather than a passive resource.

## Data Availability

The raw data supporting the conclusions of this article will be made available by the authors, without undue reservation.

## References

[B1] Avendano-Uribe B. E. Lombana-Bermudez A. Flórez L. V. Chaparro E. Hernandez-Morales A. C. Archbold J. . (2022). Engaging scientific diasporas in STEAM education: the case of science clubs Colombia. Front. Res. Metr. Anal. 7:898167. doi: 10.3389/frma.2022.898167 35837664 PMC9274008

[B2] Bonilla K. Echeverría-King L. F. Serafim M. Mabotha T. Buyuktanir Karacan D. (2023). Editorial: engaging scientific diasporas for development: policy and practices. Front. Res. Metr. Anal. 7:1102805. doi: 10.3389/frma.2022.1102805 36686409 PMC9848732

[B3] Bonilla K. Romero-Oliva C. S. Arrechea S. Ortiz Osejo N. Y. Mazariegos S. Alonzo M. . (2022). Engaging the Guatemala scientific diaspora: the power of networking and shared learning. Front. Res. Metr. Anal. 7:897670. doi: 10.3389/frma.2022.897670 35755144 PMC9215311

[B4] Diez E. Freites Y. García-Pérez M. Ordóñez L. Pineda J. Requena J. . (2020). Venezuelan Research Community Migration: Impacts and Public Policy Implications. Washington, DC: Inter-American Development Bank. doi: 10.18235/0002776

[B5] Echeverría King L. Piñeros-Ayala R. E. Pantović B. Flores-Zamora A. F Figueroa P. (2024). “A Taxonomy of Science Diplomacy From a Latin American and Caribbean Perspective,” in Developments and Approaches in Science Diplomacy: Latin America and the Caribbean (Hershey, PA: IGI Global).

[B6] Echeverría-King L. Pantović B. Bonilla K. Moreno García D. (2025). Bridging Knowledge Frontiers: A Comparative Analysis of International Strategies and Methodologies to Engage Scientific Diasporas and Foster Transnational Collaboration. Barranquilla: Universidad Simón Bolívar/International Development Research Centre (IDRC). doi: 10.17081/r.book.2025.10.17024

[B7] Ferreira G. G. C. Gimenez A. M. N. (2025). Policies on diaspora in Brazil. Barranquilla: Universidad Simón Bolívar/International Development Research Centre (IDRC). doi: 10.17081/r.report.2025.11.17129

[B8] Gómez-Flores P. Morales-Salgado V. Maza A. Villarreal A. Lara-Jacobo L. R. Jiménez-Córdova M. I. . (2022). Mexican scientist diaspora in North America: a perspective on collaborations with México. Front. Res. Metr. Anal. 7:898896. doi: 10.3389/frma.2022.898896 35719276 PMC9200957

[B9] Hernández Mondragón A. C. Cuapio A. (2025). Engaging the Scientific Diaspora: The Case of Mexico. Barranquilla: Universidad Simón Bolívar/International Development Research Centre (IDRC). doi: 10.17081/r.report.2025.11.17124

[B10] Jarquín-Solís M. E. (2022). Diseñar Puentes con la Diáspora Científica de un País: Soluciones Desde la Academia. Revista ESAL. Available online at: https://www.academia.edu/66041872/Dise%C3%B1ar_puentes_con_la_di%C3%A1spora_cient%C3%ADfica_de_un_pa%C3%ADs_Soluciones_desde_la_academia (Accessed Abr. 01, 2026).

[B11] Jarquin-Solis M. E. Lin-Shiao E. Guerra M. Calderón Zúñiga K. Mora Solórzano D. Gutiérrez J. M. (2022). Voices of the Costa Rican scientific diaspora: policy lessons from a decade of experiences from our scientists abroad. Front. Res. Metr. Anal. 7:904029. doi: 10.3389/frma.2022.904029 36570595 PMC9773386

[B12] Meyer J.-B. Brown-Luthango M. (1999). Scientific diasporas: a new approach to the brain drain. Discussion Paper Series - No. 41, MOST Programme UNESCO. Available at: https://www.researchgate.net/publication/237291192_Scientific_Diasporas_A_New_Approach_to_the_Brain_Drain (Accessed March 05, 2026).

[B13] Meyer J.-B. Wattiaux J.-P. (2006). Diaspora knowledge networks: vanishing doubts and increasing evidence. Int. J. Multicult. Soc. 8, 4–24

[B14] Pantović B. Michelini G. (2018). Ciencia y cultura de la memoria en la diplomacia serbia. Rev. CIDOB d'Afers Int. 120, 241–258. doi: 10.24241/rcai.2018.120.3.241

[B15] Piñeros Ayala R. (2025). The Colombian Scientific Diaspora: Creating Bridges of Knowledge. Barranquilla: Universidad Simón Bolívar/International Development Research Centre (IDRC). doi: 10.17081/r.report.2025.11.17130

[B16] Pombo K. (2025). Engagement of the Argentine Scientific Diaspora: Analysis of the RAICES Program Network of Argentine Researchers and Scientists Abroad. Barranquilla: Universidad Simón Bolívar/International Development Research Centre (IDRC). doi: 10.17081/r.report.2025.11.17126

[B17] Prieto J. Scott C. A. (2022). Scientific diasporas and the advancement of science diplomacy: the InFEWS US- China program in the face of confrontational “America First” diplomacy. Front. Res. Metr. Anal. 7:944333. doi: 10.3389/frma.2022.944333 36277735 PMC9583381

[B18] UNHCR (2025). Venezuela situation. United Nations High Commissioner for Refugees. Available online at: UNHCRVenezuela Situation page (Accessed June 01, 2026).

